# Mild Cognitive Impairment in Parkinson’s Disease—What Is It?

**DOI:** 10.1007/s11910-018-0823-9

**Published:** 2018-03-10

**Authors:** Rimona S. Weil, Alyssa A. Costantini, Anette E. Schrag

**Affiliations:** 1 Dementia Research Centre, UCL, London, UK; 2 Department of Neurodegeneration, UCL, London, UK; 3 Department of Clinical Neuroscience, UCL, London, UK; 4 UCL Institute of Neurology, Rowland Hill Street, NW3 2PF, London, UK

**Keywords:** Parkinson’s disease, Mild cognitive impairment, Dementia

## Abstract

**Purpose of Review:**

Mild cognitive impairment is a common feature of Parkinson’s disease, even at the earliest disease stages, but there is variation in the nature and severity of cognitive involvement and in the risk of conversion to Parkinson’s disease dementia. This review aims to summarise current understanding of mild cognitive impairment in Parkinson’s disease. We consider the presentation, rate of conversion to dementia, underlying pathophysiology and potential biomarkers of mild cognitive impairment in Parkinson’s disease. Finally, we discuss challenges and controversies of mild cognitive impairment in Parkinson’s disease.

**Recent Findings:**

Large-scale longitudinal studies have shown that cognitive involvement is important and common in Parkinson’s disease and can present early in the disease course. Recent criteria for mild cognitive impairment in Parkinson’s provide the basis for further study of cognitive decline and for the progression of different cognitive phenotypes and risk of conversion to dementia.

**Summary:**

Improved understanding of the underlying pathology and progression of cognitive change are likely to lead to opportunities for early intervention for this important aspect of Parkinson’s disease.

## Introduction

Mild cognitive impairment (MCI) is a concept introduced in the 1980s to characterise mild cognitive deficits that did not amount to a diagnosis of dementia [1], in the context of Alzheimer’s disease. More recently, this concept has been used in the context of Parkinson’s disease (PD), where cognitive deficits are common even at the point of diagnosis. However, there is considerable controversy on the use, definition, assessment and prognostic value of this concept. Here, we review current understanding of MCI in Parkinson’s disease from the point of view of epidemiology, recently described definitions, underlying pathophysiology, detection methods and tracking the presence of cognitive involvement in PD and ongoing controversies in this area.

## Why Does MCI Matter?

Dementia affects 50% of patients with Parkinson’s within 10 years of diagnosis [2] but the timing and severity vary considerably between individuals. Identifying patients at risk of dementia and those at the earliest stages of cognitive involvement is important for three key reasons: (1) as new disease-modifying treatments in Parkinson’s are emerging [3], early intervention to slow or prevent Parkinson’s dementia is becoming a realistic prospect; (2) earlier detection of cognitive involvement offers the hope of prognostic information. This can allow an affected individual to better plan for their own future and enables policy makers and healthcare providers to plan health and social needs for the population; (3) finding the earliest features of cognitive involvement may provide insights into underlying mechanisms of disease progression, ultimately leading to identification of novel therapeutic targets.

## MCI in Individuals Without PD

MCI has been defined as a syndrome of cognitive decline that is greater than would be expected for an individual’s age and level of education that does not interfere with that individual’s ability to perform activities of daily life [4]. It is distinct as an entity from dementia, where cognitive difficulties are greater and have an impact on day-to-day functioning. The concept of MCI was described in the nineteenth century by Prichard [5] who noted that the earliest sign of dementia was loss of recent memories. Some degree of cognitive deficits were thought to be a normal part of ageing and various terms were used to describe this, including age-associated memory impairment, age-associated cognitive decline and benign senescent forgetfulness [6, 7]. With the advent of new scales to measure subjective and objective cognition, such as the Clinical Dementia Rating and the Global Deterioration Scale for Ageing and Dementia, in the 1980s, an intermediate phase between normal ageing and dementia became more widely recognised [8, 9]. At that point, it was defined as the presence of subtle deficits in cognition with some impairment in executive function. Following an expert international conference, the diagnosis and management of MCI was clarified [4] and later revised [10].

The prevalence of MCI is estimated to be 3–19% in the elderly population [11]. Conversion rates to dementia vary depending on the setting [12], with 11–33% conversion over 2 years. Notably, in a community setting, 44% of people with MCI were shown to return to normal after 1 year [11]. In contrast, higher rates of conversion to dementia are seen in patients tested in memory clinics, with estimates of 16–18% per year [13, 14]. Memory-led or amnestic MCI is particularly predictive of dementia, with rates as high as 41% after 1 year and 64% after 2 years for amnestic MCI [8, 15, 16]. Impairment of delayed recall is a strong predictor of progression to Alzheimer’s disease in longitudinal studies [17, 18]. Patients with MCI in this context show quantitative differences from patients with no evidence of cognitive impairment, with reduced medial temporal lobe volumes [19, 20] and changes on PET imaging [21, 22]. In the field of Alzheimer’s research and therapeutics, there is now pressure to intervene early to prevent the development of dementia and to start treatment in patients with MCI. However, clinical trials thus far have been disappointing [23].

## MCI in PD

Cognitive deficits are common in PD, even early in the disease when there may be no cognitive complaints [2]. The concept of mild cognitive impairment in Parkinson’s disease (PD-MCI) is therefore emerging as an intermediate stage between normal cognition and dementia, similar to amnestic MCI in AD.

## Epidemiology

Epidemiological studies report prevalence rates of MCI in PD of 25–50% of patients [24–26], depending on the population and clinical setting. It is estimated to be as high as 20–42% at the time of diagnosis [25, 27, 28••].

## PD-MCI Phenotypes

PD-MCI is a heterogeneous entity in phenotype, timing and progression, with a range of cognitive domains affected [28••]. Characteristically, where a single domain is involved, this is mostly a non-amnestic subtype [29, 30•]. However, subtypes with predominant deficits in attention, memory, executive function, psychomotor speed and visuospatial abilities have also been described [24, 26, 27, 31], and it frequently involves deficits across multiple domains [32–34].

One influential model for the pattern of cognitive deficits in PD was framed by Barker and colleagues as the dual syndrome hypothesis [35]. In their longitudinal CamPaIGN study [2, 36], they observed two distinct patterns of cognitive involvement in PD: a fronto-striatal/executive group and a posterior cortical/visuospatial cognitive phenotype [36]. One challenge, when comparing performance across cognitive domains, is maintaining the same level of difficulty across domains. Indeed, the relative preservation of visuospatial abilities in patients in some studies [27, 32] may be a result of the lack of sensitivity of the tests used, compared with more difficult tests of memory.

## PD-MCI—New Proposed Definition

The definition of PD-MCI has varied between different studies, prompting a task force from the International Parkinson and Movement Disorder Society (MDS) to provide a unified definition based on a literature review and expert consensus [28••].

According to these new criteria (see Table 1), in patients with a diagnosis of PD, PD-MCI is defined as an insidious decline in cognitive abilities reported by patient or informant or observed by the clinician, not caused by other comorbidities. In contrast to dementia, cognitive deficits are present on testing but do not interfere with functional independence of the patient [28••].

**Table 1:** Criteria for the diagnosis of PD-MCI

Inclusion criteria: Diagnosis of Parkinson’s disease as based on the UK PD Brain Bank Criteria Gradual decline, in the context of established PD, in cognitive ability reported by either the patient or informant, or observed by the clinician Cognitive deficits on either formal neuropsychological testing or a scale of global cognitive abilities Cognitive deficits are not sufficient to interfere significantly with functional independence, although subtle difficulties on complex functional tasks may be present
Exclusion criteria: Diagnosis of PD dementia based on MDS Task Force proposed criteria Other primary explanations for cognitive impairment (e.g. delirium, stroke, major depression, metabolic abnormalities, adverse effects of medication or head trauma) Other PD-associated comorbid conditions (e.g. motor impairment or severe anxiety, depression, excessive daytime sleepiness or psychosis) that, in the opinion of the clinician, significantly influence cognitive testing
PD-MCI level guidelines: A. Level I (abbreviated assessment) • Impairment on a scale of global cognitive abilities validated for use in PD *or* • Impairment on at least two tests, when a limited battery of neuropsychological tests is performed (i.e. the battery includes less than two tests within each of the five cognitive domains, or less than five cognitive domains are assessed) B. Level II (comprehensive assessment) • Neuropsychological testing that includes two tests within each of the five cognitive domains (i.e. attention and working memory, executive, language, memory and visuospatial) • Impairment on at least two neuropsychological tests, represented by either two impaired tests in one cognitive domain or one impaired test in two different cognitive domains • Impairment on neuropsychological tests may be demonstrated by any one of the following: ○ Performance approximately 1 to 2 SDs below appropriate norms ○ Significant decline demonstrated on serial cognitive testing ○ Significant decline from estimated premorbid levels
Subtype classification for PD-MCI (optional, requires two tests from each of the five cognitive domains assessed and is strongly suggested for research purposes): PD-MCI single-domain—abnormalities on two tests within a single cognitive domain (specify the domain), with other domains unimpaired or PD-MCI multiple-domain—abnormalities on at least one test in two or more cognitive domains (specify the domains)

From Litvan et al. (2012, with permission

In view of the varying availability of neuropsychological testing in different clinical settings, the Task Force proposed two levels of definition: level I uses a pragmatic approach that allows for the diagnosis of PD-MCI based on an abbreviated cognitive assessment, either with a global scale such as the Montreal Cognitive Assessment (MoCA) or a limited range of neuropsychological tests. Within this level of definition, impairment must be present on a scale of global cognitive abilities or at least two out of alimited battery of neuropsychological tests.

Level II definition of PD-MCI is based on a more comprehensive assessment with at least two tests for each of the five cognitive domains (attention and working memory, executive, language, memory and visuospatial) and allows more accurate assessment. A range of suitable standard neuropsychological tests can be used for this assessment [28••]. Deficits need to be detected on at least two tests for each of the five cognitive domains. This can be either two tests within one cognitive domain or two tests across different cognitive domains. Impairment is defined as 1 to 2 standard deviations below population norms, a significant decline from previous testing or a significant decline from the individual’s estimated premorbid level.

Further subtyping of PD-MCI by the task force is designed to enable future research into whether impairments in specific cognitive domains have distinct neurobiological substrates. Using this framework, PD-MCI can be divided into single or multiple domains, according to involvement of deficits on two tests within or across cognitive domains, respectively. Importantly, the task force recommended reporting exactly which cognitive domains are affected to enable differences between the subtypes to be studied.

## Conversion from MCI to Parkinson’s Disease Dementia

Generally, the presence of PD-MCI predicts the development of PD dementia (PDD) [28••], but longitudinal studies show heterogeneity in patterns of conversion to PDD and a notable proportion of patients with PD-MCI will revert to normal cognition during follow-up. In longitudinal studies, rates of conversion from PD-MCI to PDD are broadly similar: 62% after 4-year follow-up [37], compared with only 20% in patients without cognitive involvement; 50% at 5 years in a community-based Swedish study [38]; and 39% at 5-year follow-up in the Norwegian ParkWest study [39•]. Notably, rates of reversion back to normal cognition were 11% in the Swedish study [38] and 28% in the ParkWest study [39•]. The variability of these results is likely to reflect differences in study populations, assessment methods and definitions. The level II MDS PD-MCI definition as a predictor of PDD was recently validated in a longitudinal study, where conversion rates from PD-MCI to PDD varied depending on degree of cognitive deficit at baseline [40]: patients scoring between 1 and 1.5 SD below the mean in at least two cognitive tests had 12% rate of conversion to PD dementia during follow-up, compared with patients who fell below 2 SD from the mean (in two tests) at baseline, of whom 50% developed PD dementia after follow-up.

## MCI in Prodromal PD

Recent reports show that cognitive deficits, especially in fluency, can already be detected in the prodromal phase of PD, even before the onset of motor symptoms. Poor cognitive function, measured using letter-digit substitutions, Stroop colour test, verbal fluency and 15-word list learning tests, is associated with increased risk of developing PD [41••]. Cognitive deficits are even seen in people who do not yet have Parkinson’s disease but are known to be at risk (e.g. due to hyposmia or REM sleep disorder [42, 43] and in the Parkinson’s at Risk study, unaffected relatives of people with Parkinson’s disease who had hyposmia and abnormal DaT scans also showed impaired performance in tests of attention, verbal fluency and processing speed [44].

## How to Determine Who Is at Risk

### Demographic and Clinical Risk Factors

Clinical factors associated with a higher risk of cognitive involvement in PD include the following: older age [38, 39] at diagnosis (age over 70 associated with odds ratio (OR) 5.2 in one study [45], akinetic rigid phenotype (freezing and/or falls, OR 1.8 [45], poor performance on verbal fluency tests and higher rates of comorbidity [2]. REM sleep behavioural disorder (OR 5.4 [45] and dysautonomia (OR 5.3 for systolic blood pressure drop [45]) are also associated with higher risk of developing dementia in PD [45, 46]. Other associated factors include male gender (OR 4.1 [45] and non-motor symptoms including depression and anxiety [47]. Lower apathy scores and higher Epworth Sleepiness scores were relatively protective, being associated with reversion to normal cognition at follow-up [39•]. Conversely, other studies have shown increased risk of PDD with excess daytime sleepiness [48]. Whether those that reverted to normal cognition had other causes of cognitive deficits will need to be tested in longitudinal studies.

Higher rates of conversion from PD-MCI to dementia are associated with, older age, depression and a non-tremor dominant phenotype [2, 37, 39•]. Consistent neuropsychological profiles of PD-MCI associated with higher conversion rates to dementia are non-amnestic MCI (affecting domains other than memory) [37] including deficits in fluency, mental flexibility and visuospatial domains, although memory deficits are also reported [2, 38]. The two groups defined by Barker et al. also differed in their rates of conversion to dementia: those with a frontal–executive phenotype had a higher rate of reversion to normal cognition than those with visuo-perceptual deficits [2, 35]. As definitions of PD-MCI are increasingly refined and improved, more accurate predictions of the risk of development of dementia in PD based on cognitive profiles of PD-MCI are likely to be developed.

### Biomarkers

Biomarkers, such as CSF proteins and imaging, are not currently part of the definition of PD-MCI, although they do form components of criteria for non-PD MCI [10]. They may become important in the future as our understanding of PD-MCI increases and as clinical trials to slow the progression of dementia in PD are developed.

### CSF

Lower levels of CSF amyloid beta may reflect brain amyloid deposition [49]. Decreased CSF amyloid beta is found in patients with PDD and PD [50, 51] and correlates with scores in verbal learning and Stroop word colour tests [52]. CSF amyloid beta also correlates with risk of developing dementia in PD [53–55]. Importantly, lower levels of CSF amyloid beta are also seen in patients with PD before they develop PD-MCI and are therefore predictive of cognitive involvement in PD [54]. However, levels of amyloid beta are not lower in patients with PD-MCI than those with normal cognition in PD [55, 56]. This may reflect the wide definition of PD-MCI and its relative lack of specificity for future conversion to PDD.

The story is less clear for tau. Higher CSF tau is correlated with impaired performance on cognitive tests of naming and memory when tested in mixed groups of patients with PD and PDD [50, 53], and CSF tau is slightly higher in patients with PD and cognitive involvement (33/36 of whom had PD-MCI) [56]. But other studies in patients at earlier disease stages have showed no associations between CSF tau and cognition in PD [57].

### Radioligand Imaging

Radioligand imaging can provide insights into mechanisms of cognitive involvement in PD-MCI and may have a potential role as a clinically useful biomarker of MCI in PD. Patients with PD-MCI show hypometabolism in posterior brain regions compared with PD patients with no evidence of cognitive involvement [58] and reduced metabolism is seen using FDG-PET in posterior cortical regions in PD patients who later developed PD dementia after 6 years of follow-up [59, 60].

Reduced dopamine transporter uptake correlates with executive function [61, 62], but executive dysfunction does not always progress to dementia in PD [63]. More recently, reduced caudate uptake on DAT-SPECT imaging was shown to predict cognitive decline in PD, especially when combined with other measures including age and CSF [64]. Finally, ligands binding to amyloid may have a role in detecting PD-MCI as amyloid binding negatively correlates with cognition in PD [65, 66] and increased amyloid binding at baseline increases the likelihood of cognitive involvement in PD [65].

### MRI

MRI structural differences in grey matter between patients with PD-MCI and patients without cognitive involvement show varying patterns of atrophy involving all cortical brain areas [67–69], as well as subcortical regions [68, 70, 71]. These differences are likely to be partly due to variation in MRI methodology to assess cortical thickness as well as differences in cognitive tests. However, a more fundamental reason for differences in findings is due to the low sensitivity of grey matter atrophy in detecting cognitive involvement in PD. Grey matter atrophy indexes neuronal cell death which is a relatively late event in PD dementia. Therefore, in order to detect the earliest signs of PD-MCI, measures that are sensitive to earlier pathological events are needed. These are likely to be techniques such as diffusion tensor imaging that detect axonal and synaptic changes [72] which occur at earlier stages in PD-MCI.

Diffusion-weighted imaging MRI techniques [73, 74] that provide information about white matter integrity are sensitive to axonal damage. Estimates of mean diffusivity (MD) and fractional anisotropy (FA) index the overall displacement of molecules and the pattern of restriction of diffusion of molecules, respectively. Indeed, white matter changes increase as cognition worsens in PD, as measured using FA and MD [73–75]. When measured concurrently, white matter alterations are seen in patients with PD *before* changes in grey matter atrophy [72, 74, 76]. New techniques for quantification of structural change across the entire network are emerging that are likely to show sensitivity for changes in PD-MCI [77].

### Underlying Pathophysiology of MCI-PD

#### Alpha Synuclein

The precise pathophysiological mechanisms underlying cognitive involvement in PD are still not fully understood. There is only scarce data specific to PD-MCI, rather than dementia in PD. This is partly due to the fact that PD-MCI occurs relatively early in the disease, with only limited series of PD-MCI cases seen at post mortem (see [78] for review). It is becoming clear that PD-MCI is likely to be characterised by a combination of underlying pathologies [79, 80]. Lewy bodies (intracellular inclusions made up of alpha synuclein) are classically associated with PD and PDD [81, 82]. However, it is the *combination* of Lewy bodies with Alzheimer’s pathology (fibrillary beta amyloid and intraneuronal tangles of hyperphosphorylated tau) that is most strongly associated with dementia in Parkinson’s disease [83–85]. Indeed, there seems to be synergistic effect between alpha synuclein and beta amyloid pathology. For example, a recent study showed a strong correlation between the extent of neurofibrillary tangles and alpha synuclein [86•], and this observation is supported by mouse studies and in vitro model systems [87, 88].

#### Location of Pathological Accumulations

As well as accumulation of pathological proteins, the morphological characteristics and location of pathological accumulations within affected cells appear to be critical in patients with cognitive involvement in PD. The synapse may be important in the earlier stages of cognitive involvement in PD. The physiological form of alpha synuclein localises to the presynaptic terminal, and in both PD and DLB, alpha synuclein aggregates localise to the synapse [89, 90]. Consistent with this, reduced levels of two synaptic proteins (neurogranin and SNAP25) and neocortical ZnT3, a protein involved in synaptic zinc regulation, are all associated with cognitive involvement in PD [91, 92].

Axonal involvement also appears to be an important early feature of PD, with alpha synuclein accumulation starting in the axonal compartment, before neuronal loss is seen [93]. Moreover, neurones that are preferentially affected in PD-associated dementia show extreme length. For example, the cholinergic cells of the nucleus basalis of Meynert and the serotonergic cells of the raphe nucleus are both implicated in PDD and both show long, thin and complex branching axons [94–97].

#### Neurotransmitters

Changes in the levels of specific neurotransmitters may also have an important role in PD-MCI. There is a correlation between neuronal loss in the nucleus basalis of Meynert, cortical cholinergic deficits and the degree of cognitive impairment in PD [94]. Reduced choline acetyltransferase activity in frontal and temporal regions correlates with Lewy body load and with cognitive deficits [98]. In PD patients without dementia, cholinergic denervation is associated with impaired verbal learning and Stroop performance [99, 100]. These histopathological and neuroimaging findings are borne out by the observation that anticholinergic drugs that are used to treat motor symptoms in PD impair both memory [101] and frontal executive function [102]. Furthermore, treatment with cholinesterase inhibitors, which restore levels of acetyl choline [103], improve cognitive function in PDD and DLB, lending further support to the role of cholinergic neurotransmission in these conditions.

Noradrenergic pathways may also be involved in cognitive changes in PD. As well as early loss of peripheral noradrenergic neurones in PD [104], noradrenaline exerts central effects, possibly by integrating activity across brain regions [105]. Alpha synuclein deposition is particularly heavy in the locus coeruleus, the chief source of noradrenergic projections in the brain [106]. This may form the basis of orthostatic hypotension, commonly seen in PD with associated cognitive deficits.

The relationship between dopamine and cognition is less clear-cut. Dopamine has beneficial effects on tasks sensitive to frontal lobe dysfunction [101, 107, 108] and uptake of dopamine transporter ligands may predict change in cognitive function in early PD [64]. However, visuospatial and memory tasks are not affected by dopaminergic medications [109], and a recent therapeutic trial of rasagaline that targets dopaminergic pathways did not show improved cognition in patients with PD-MCI [110].

In summary, cognitive involvement in PD-MCI is likely to arise from a combination of mechanisms. Pathological accumulation of alpha synuclein as well as tau and amyloid beta are of importance, especially within neurones with particularly long axons, and neurotransmitter changes especially involve loss of cholinergic function.

## Current Challenges and Controversies

### DLB and PDD

There is a longstanding debate as to whether PD dementia and dementia with Lewy bodies (DLB) are one or distinct entities [111]. Parkinson’s disease dementia is defined as progressive cognitive decline in the context of established PD at least 1 year after onset of Parkinsonian motor symptoms [112]. Conversely, dementia occurring before, simultaneous to or within the first year of Parkinsonism is DLB [113]. The presence of cognitive deficits in prodromal and preclinical PD calls into question the very definition of DLB. If cognitive changes can be detected years before the onset of classical PD, this suggests that a cognitive phenotype is core to the diagnosis of PD itself and blurs the boundaries with DLB. This issue remains unresolved, although it is acknowledged in the current Movement Disorder Society criteria for both DLB and PD-MCI [28••, 113]. As our understanding of the underlying mechanisms of cognitive involvement in Parkinson’s disease increases, definitions of DLB, PDD and PD-MCI are likely to become clarified.

### PD-Associated Comorbidities

Other factors commonly associated with PD may impact on cognition as well as general levels of alertness and attention. These include effects of medications, mood changes such as depression and apathy, as well as sleep disorders. Motor fluctuations can also influence cognition and an individual’s global abilities in day-to-day functioning. These should all be taken into consideration when assessing patients for potential PD-MCI. Where possible, therapeutic interventions should be targeted to optimise each of these factors.

### Heterogeneity in PD-MCI

As currently defined, PD-MCI is a heterogeneous entity, encompassing patients with deficits in any cognitive domain, and with varying levels of impaired functioning. Evidence already suggests that some sub-groups, particularly those with deficits in visuospatial performance, are at the highest risk of developing PD dementia [2, 35]. Maintaining wide definitions, with poorly differentiated cognitive profiles, prevents accurate comparisons across studies. Critically, whilst these boundaries are blurred, clinically useful disease stratification will not be possible. There is clearly a need for better cognitive phenotyping to enable well-defined sub-groups that are more likely to show similar rates of disease progression. In part, this could be improved with more sensitive and more widely available measures of visuospatial function that are beginning to emerge [114].

### Therapies to Treat PD-MCI

#### Pharmacological

There are currently no pharmacological treatments to improve cognition specifically in PD-MCI, although this is now an area of active research. A recent small cross-over study of the rivastigmine patch reported a trend for [115] improvement in the primary outcome measure of global improvement [116]. Recent trials of atomoxetine, a selective noradrenaline reuptake inhibitor, suggested improvement in global cognition in patients with depression in PD [117] as well as improved decision-making, attention and planning in patients with PD without dementia [118] supporting the potential role for noradrenergic pathways in PD-MCI.

Trials of disease-modifying therapies in Parkinson’s are currently underway, with the ultimate aim of slowing the disease. Targets include the lysosomal pathway [119] and immunotherapies targeted against alpha synuclein [120] as well as repurposing of established drugs [3]. Whether these treatments will have specific effects on slowing cognitive involvement in PD or in PD-MCI will need to be tested.

#### Non-Pharmacological

Despite a large number reports of cognitive training to improve cognition in PD, the impact of these interventions is still not clear. A recent meta-analysis of seven studies, including 272 participants [121], found key methodological problems. For example, 6 out of 7 studies suffered from bias due to lack of adherence to intention to treat analyses, and two out of 7 did not blind assessors. They concluded that the body of evidence is small but that there may be a small overall effect on cognition. They found no improvement in scores such as the MMSE or MoCA and that most gains were in executive skills such as planning. Notably, memory and visuospatial domains did not show significant improvement.

Interventions using physical exercise show more promise in improving cognitive outcomes in PD. Across several studies, moderate intensity aerobic exercises performed 2–3 times per week lead to some improvement in executive function [122–126] as well as language function [127]. Large-scale RCTs are now underway [128] and will be of critical importance to assess the effectiveness of these physical interventions in preventing or slowing cognitive involvement in PD and PD-MCI.

## Conclusions

PD-MCI as a clinical entity has an important role in understanding the progression of PD both at an individual and a population level. Future work will require more longitudinal studies of the progression of cognitive change in PD, with more sensitive visuospatial measures. It is likely that definitions and biomarkers will begin to incorporate multimodal measures alongside neuropsychological tests. Recognising the earliest stages of cognitive involvement will allow disease stratification and personalised treatment, with the potential for early intervention. It will enable better-powered clinical trials, and potential outcome measures, ultimately to develop treatments to prevent the progression of dementia in PD.

## Abbreviations

MCI: Mild cognitive impairment
MOCA: Montreal cognitive assessment
PD: Parkinson’s disease
PDD: Parkinson’s disease dementia
PD-MCI: Mild cognitive impairment in the context of Parkinson’s disease
